# Ophthalmic Disorders in Adults with Down Syndrome

**DOI:** 10.1155/2012/974253

**Published:** 2012-04-18

**Authors:** Sharon J. Krinsky-McHale, Edmund C. Jenkins, Warren B. Zigman, Wayne Silverman

**Affiliations:** ^1^Department of Psychology, New York State Institute for Basic Research in Developmental Disabilities, 1050 Forest Hill Road, Staten Island, NY 10314, USA; ^2^Department of Human Genetics, New York State Institute for Basic Research in Developmental Disabilities, 1050 Forest Hill Road, Staten Island, NY 10314, USA; ^3^Department of Behavioral Psychology, Kennedy Krieger Institute and Department of Psychiatry and Behavioral Sciences, Johns Hopkins University School of Medicine, 707 N. Broadway, Baltimore, MD 21205, USA

## Abstract

A myriad of ophthalmic disorders is associated with the phenotype of Down syndrome including strabismus, cataracts, and refractive errors potentially resulting in significant visual impairment. Ophthalmic sequelae have been extensively studied in children and adolescents with Down syndrome but less often in older adults. In-depth review of medical records of older adults with Down syndrome indicated that ophthalmic disorders were common. Cataracts were the most frequent ophthalmic disorder reported, followed by refractive errors, strabismus, and presbyopia. Severity of intellectual disability was unrelated to the presence of ophthalmic disorders. Also, ophthalmic disorders were associated with lower vision-dependent functional and cognitive abilities, although not to the extent that was expected. The high prevalence of ophthalmic disorders highlights the need for periodic evaluations and individualized treatment plans for adults with Down syndrome, in general, but especially when concerns are identified.

## 1. Ophthalmic Disorders in Adults with Down Syndrome

Down syndrome is the most prevalent genetic disorder associated with intellectual disability and is due to the presence of complete or partial triplication of chromosome 21 [1]. It is associated with a characteristic physical and cognitive phenotype, although almost every aspect of the phenotype shows variability in terms of occurrence and severity [2, 3]. Down syndrome carries with it an increased risk of congenital heart defects, hearing loss, autoimmune diseases, shortened life expectancy, early onset Alzheimer's disease, and other concerns related to health and aging that also include multiple ophthalmic disorders [4–7]. Earlier studies have indicated increased risk for abnormality in virtually all structures of the eye including the lid, iris, cornea, lens, and retina [8–11]. As a consequence, *nystagmus*, *strabismus*, *keratoconus*, *amblyopia*, *cataracts*, and *refractive errors* are prevalent in this population potentially resulting in significant visual impairment [12, 13] (see Appendix for brief definitions of italicized terms). While no specific ophthalmic disorder seems to be pathognomonic of Down syndrome, many individuals present with a combination of conditions [12, 14].

The ophthalmic sequelae in children and adolescents with Down syndrome have received considerable attention [12, 15–18], but the prevalence of vision problems in older adults has been reported less often. The life expectancy of adults with Down syndrome has increased dramatically over the last several decades [19, 20] and as a consequence, they are prone to experience health problems associated with advancing age, such as visual functioning deficits that are likely to be similar to or more severe than those seen in adults without intellectual disability. The studies that do exist on vision in adults with Down syndrome have generally found that the number and severity of ophthalmic disorders increase with age [9, 21–27]. Van Schrojenstein Lantman-de Valk et al. [23] examined the sensory functioning of older individuals with intellectual disability in the Netherlands, who were between 50 and 59 years of age and found that visual impairment occurred in 46% of adults with Down syndrome. This number increased significantly with age such that 85% of people with Down syndrome, 60 years of age and older, experienced visual impairment [9, 21, 27]. The age-specific prevalence for specific ophthalmic disorders has rarely been reported [28] although van Schrojenstein Lantman de Valk et al. [23] found that the prevalence of cataracts in adults with Down syndrome increased from 16% of individuals between 50 and 59 years of age to 63% of individuals 60 years of age and older (also see [29]). Van Buggenhout et al. [27] found that the severity of ophthalmic disorders increased with age in adults with Down syndrome. While moderate-to-severe vision loss was reported in 18% of individuals between 30 and 39 years of age, prevalence increased to 28% for individuals between 40 and 49 years of age and to almost 50% for individuals between 50 and 59 years of age. Thus, it is likely that changes in vision are among the features of atypical aging seen in individuals with Down syndrome in middle age.

Prevalence of ophthalmic disorders has been found to increase dramatically with severity of intellectual impairment in individuals with Down syndrome ([9, 30]; cf. [15]). For example, Evenhuis et al. [9] observed visual impairment in 4.5% of individuals with mild or moderate intellectual disability but in 74% of individuals with severe or profound intellectual disability. Several researchers examined the relation between severity of intellectual disability and prevalence of specific disorders [10, 31, 32]. McCulloch et al. [31] found that 25% of individuals with mild intellectual disability had strabismus compared to 60% of individuals with profound intellectual disability. Further, *esotropia *(the form of strabismus where one or both eyes tend to drift inward) was typically found in those with milder disabilities, whereas *exotropia* (where one or both eyes tend to drift outward) was most common in those with more severe disabilities [31]. Other associations with severity of intellectual impairment have been found for *visual acuity* as well as refractive errors [31, 32].

Intellectual disability results in significantly impaired functioning, but when it cooccurs with visual impairment, overall disability can be exacerbated and quality of life may be reduced. Visual impairment has been found to significantly decrease independent living skills, communication and language skills, social skills, and initiative and persistence [33, 34]. The aim of the present study was to evaluate the characteristics and prevalence of specific ophthalmic disorders in older adults with Down syndrome (from 30 to 83 year olds) and to determine if the presence of ophthalmic disorders affects adaptive behavior and cognitive status. In addition, inclusion of individuals with a wide range of intellectual disability (FSIQ range = 20–71) enabled the examination of how prevalence of ophthalmic disorders varies as a function of intellectual disability.

## 2. Method

### 2.1. Human Subject Approvals

This study was approved by the Institutional Review Boards of the New York State Institute for Basic Research in Developmental Disabilities and the Johns Hopkins University School of Medicine. Participants gave their assent for all procedures, and for each participant, an authorized representative provided informed consent.

### 2.2. Participants

The participants were 455 adults with Down syndrome, who were enrolled in a larger multidisciplinary study focused on aging and dementia (see [35, 36] for inclusion criteria).  Table 1 presents the demographic characteristics of the participants. There was a preponderance of females (69.5%), which reflects the interests and sampling procedures of our overall program, one goal of which was to investigate women's health issues and aging. Multiple IQs were obtained from clinical records and testing typically occurred when the participants were children or young adults. The specific IQ tests and dates of administration were also recorded. We generated a “consensus Full Scale/Composite IQ” for each participant using either the results actually obtained or, in cases where data were only available from the Wechsler Adult Intelligence Scale [37], an estimated “Stanford-Binet-equivalent” was calculated to address the compelling evidence that the various editions of the Wechsler Adult Intelligence Scale generate substantially higher IQs for this population compared to other assessments [38].

Down syndrome was confirmed cytogenetically for 368 (82.9%) individuals; 328 (89.1%) had full trisomy 21, 25 (6.8%) had trisomy 21 mosaicism, and 15 (4.1%) had an autosomal translocation. The families of 76 (16.7%) individuals refused consent for a blood sample, and we were unable to obtain a blood sample from another 12 individuals (2.6%). These 88 individuals were confirmed to have trisomy 21 based on phenotype.

### 2.3. Materials and Procedures

Participants were comprehensively evaluated at approximately 18-month intervals with an assessment battery that included detailed review of medical records, informant interviews, direct assessment of a variety of cognitive functions, collection of blood samples, and, for a selected subsample, a neurological examination. The primary data for this study came from the medical records of participants obtained from clinical or agency files and examined upon their entry into the study. These records were hand-searched and data regarding all diagnoses and clinically significant health problems were extracted and entered onto a standardized form following a protocol developed in conjunction with the broader research program. The form included questions pertaining to all body systems. It also included the date and course of treatment for specific conditions and demographic information. The presence or absence of specific ophthalmic disorders was examined for this report.

 As part of our longitudinal study, we examined the cognitive abilities and behavioral functioning of all study participants. For the current study, we report on measures where performance should be especially sensitive to visual processing and, for comparison, those that should be relatively independent of visual processing. The medical chart review, cognitive, and adaptive measures were collected contemporaneously. The American Association on Mental Retardation (AAMR)—Adaptive Behavior Scale (ABS-Part One) [39, 40], an informant-based assessment measuring a variety of functional domains, was used to examine adaptive competence and functional abilities. The skills examined within Part One are grouped into 10 behavior domains reflecting independent functioning, physical development, economic activity, language development, numbers and time, domestic activity, vocational activity, self-direction, responsibility, and socialization. The 10 adaptive domain scores were summed to create an overall index of adaptive functioning with a maximum possible score of 280.

 The cognitive abilities of participants were evaluated with direct testing. Measures sensitive to visual processing included the Block Design subtest of the WISC-R [41] plus a series of simpler items referred to as the Extended Block Design test [42]. Both tasks involved reproducing visual patterns from models with red and white Kohs blocks. These tests provided a measure of visuospatial organization, with performance requiring both an analysis of visual details and the synthesis of the final design. Procedures were consistent with those described in the WISC-R manual with the exception that testing always began with the simplest design, a single block, and progressed in difficulty to 2 × 2- and 3 × 3-block designs. Each trial had a time limit, and the score represents the number of designs completed successfully within that time frame. The dependent measure was the sum of the raw scores on these two tests (scaled scores were unavailable for the ages of our participants), with a maximum possible score of 78.

The Beery-Buktenica Developmental Test of Visual-Motor Integration was used to ascertain construction ability [43]. The task requires participants to copy simple figures using paper and pencil, starting with one straight line (in both a horizontal and vertical orientation) and a circle. Figures progressively increase in complexity by the addition of lines and shapes. A single summary score was generated to reflect overall performance using standard scoring procedures with a maximum possible score of 27.

 An adaptation of the McCarthy [44] Verbal Fluency Test was one of the “nonvisual” tasks included in our battery. It requires participants to name as many foods, animals, or clothes (two of these categories are administered in any given test cycle) as fast as possible within 20 s. A summary score was generated by adding the number of correct responses for the two categories.

 Another test independent of visual processing was a modified version of the Selective Reminding Test [45, 46]. Eight items from a single semantic category (animals or foods) are presented verbally followed by 6 trials of free recall. After the first trial, only those items that were not recalled on an immediately preceding trial are represented for learning on the next trial. The Selective Reminding Test generates multiple scores that reflect the efficiency of various memory processes [45, 47], but our primary measure of interest was the total number of items recalled over the 6 trials with a maximum score of 48.

SYSTAT 12 was used for all analyses. Chi-square analyses were conducted on categorical data. Graphic analyses were conducted on these data to determine overall significance for the set of dependent variables following procedures similar to those described by Schweder and Spjøtvoll [48]. This strategy avoids the substantial loss of power associated with a straightforward Bonferroni correction for multiple tests yet addresses concerns associated with potential inflation of type-I error probability. The General Linear Model module was used for analyses of continuous data.

## 3. Results

It was exceedingly common for older adults with Down syndrome to have an ophthalmic disorder. The medical records of 77.6% (353 of 455) adults with Down syndrome indicated they had at least one ophthalmic disorder. We found an association between age and the prevalence of having at least one ophthalmic disorder such that, as a group, individuals having an ophthalmic disorder were 2.5 years older than those who did not, *F* (1,454) = 8.35, *P* = .004. The association between sex and the prevalence of having at least one ophthalmic disorder was not significant, *χ* ^2^ (1, *N* = 455) < 1.

Data regarding the prevalence of specific ophthalmic disorders are summarized in Table 2. A wide variety of ophthalmic disorders was noted in participants' medical charts.

Cataracts were the most frequent ophthalmic disorder reported for adults with Down syndrome, affecting 191 of 455 (42%) individuals. Refractive errors were the second most frequent disorder, reported for 115 adults (25%), with *astigmatism* and *myopia* as the leading causes. Strabismus was reported in 21.1% and *presbyopia* in 12.5% of adults with Down syndrome. Legal blindness was reported in 7.7% of adults with Down syndrome. Keratoconus and nystagmus have been reported in previous studies as conditions frequently causing visual impairment in individuals with Down syndrome but were only noted in 2.9% and 3.5% of individuals in our study, respectively. *Blepharitis* and *conjunctivitis,* two inflammatory conditions of the eye that are unrelated to visual impairment, were reported for 10.1% and 13.4% of our sample, respectively. All other eye conditions were reported in small numbers.

Several disorders showed an association with age. Cataracts were more common for the older individuals, *F* (1,453) = 24.83, *P* < .001, while astigmatism, *F* (1,453) = 13.16, *P* < .001 and refractive errors, *F* (1,453) = 12.05, *P* < .001 were more frequently reported for younger individuals. The presence of all other ophthalmic disorders were found to be unrelated to age.

### 3.1. The Prevalence of Ophthalmic Disorders and the Severity of Intellectual Disability

Overall, the prevalence of having at least one ophthalmic disorder was not significantly different among intellectual disability severity groups for adults with Down syndrome. With one exception, this was also our finding for specific ophthalmic conditions. Individuals who were legally blind were more likely to have profound intellectual impairment (24 out of 33 legally blind participants) compared to their peers who were not legally blind, *χ* ^2^ (3, *N* = 436) = 34.11, *P* < .001.

### 3.2. Cataracts

Because prevalence of cataracts was high, we examined cumulative incidence by age and treatment plans for individuals with this condition. Two individuals had congenital cataracts. Congenital cataracts are considered to be a distinct phenomenon, and the two affected individuals were, therefore, excluded from these analyses.

The average age in which an individual with Down syndrome was diagnosed with cataracts was 48.43 years (SD = 9.87). Prevalence of cataracts was unrelated to intellectual disability severity, but was related to age, as discussed previously.  Table 3 presents summary prevalence data for each 10-year age interval for individuals with Down syndrome and the US national estimates for the general population (see [49]). Clearly, prevalence is higher for adults with Down syndrome, who are in their 40 s through 60 s, *χ* ^2^ (2, *N* = 455) = 1246, *P* < .10^−6^.

**Figure 1:**
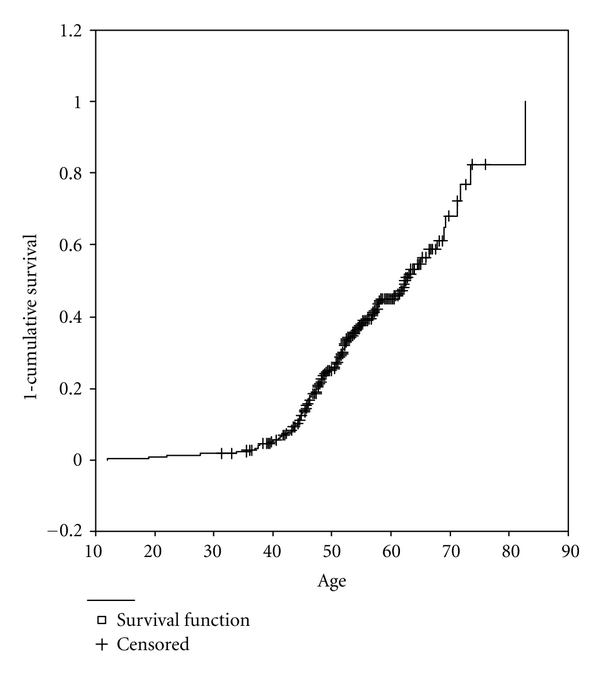
A Kaplan-Meier survival analysis stratified by age of cumulative incidence of cataracts for participants with Down syndrome.

A reconstructed cohort design [50] was used to estimate cumulative incidence of cataracts, in which each participant was considered to be at risk from birth until their current age (if unaffected) or until the age at which they received a diagnosis. A Kaplan-Meier Survival Analysis was used to estimate time-to-diagnosis.  Figure 1 clearly shows that risk for individuals with Down syndrome increased with age quite rapidly beginning at approximately 40 years of age.

#### 3.2.1. Treatment Plans

 A number of treatment options were prescribed, typically dependent on the resulting degree of vision loss experienced by an individual. The most frequent treatments included: (a) surgery with intraocular lens implantation, (b) an increase in prescription strength of glasses, or (c) surveillance for increasing vision loss.

 For almost half of the individuals with diagnosed cataracts, no treatment was undertaken at the time of diagnosis (45.3%). Typically a comment was noted in the medical record that the condition was in the early stages and was not sufficiently advanced to warrant surgery, along with a recommendation for reexamination to evaluate disease progression. Cataract surgery was reported for a relatively small number of individuals with the condition, 15.6%. For 11% of adults with Down syndrome, a change in eyeglass prescription was ordered at the time of diagnosis. The medical charts of 22.9% of individuals did not specify any treatment at the time of diagnosis.

### 3.3. Effects of Ophthalmic Disorders on Cognitive and Adaptive/Behavioral Function

To determine the impact of ophthalmic disorders on adaptive behavior and cognition, individuals with and without ophthalmic disorder(s) were compared on a number of performance measures dependent on visual processing (AAMR Adaptive Behavior Scale, the Block Design Test, and the Beery-Buktenica Developmental Test of Visual-Motor Integration) and those that were independent of visual processing (the Verbal Fluency Test and the Selective Reminding Test).  Table 4 presents the means for these measures as a function of ophthalmic status. We excluded cases where blepharitis, conjunctivitis, and/or dry eye were the only conditions reported, reasoning that they do not usually cause impairment in visual functioning. An analysis of covariance was conducted where ophthalmic status (with and without an ophthalmic disorder(s)) was the between-subjects measure and IQ was the covariate. (The number of participants that completed each test differed between tests and therefore degrees of freedom varied as well.) In adults with Down syndrome, scores on measures that relied on visual processing were related to overall ophthalmic status, although the effect sizes were small (AAMR Adaptive Behavior Scale, *F* (1,425) = 3.95, *P* = .048, Cohen's *d*′ =  .193; the Block Design Test, *F* (1,322) = 7.84, *P* = .005, Cohen's *d*′ =  .312; the Beery-Buktenica Developmental Test of Visual-Motor Integration, *F* (1,338) = 3.95, *P* = .048, Cohen's *d*′ =  .216). We next examined the effects of specific ophthalmic disorders expected to have the most substantial effects on quality of life. Being legally blind had a detrimental effect on adaptive behavior, visuospatial organization, and construction ability although the effect sizes were small (AAMR Adaptive Behavior Scale, *F* (1,425) = 4.28, *P* = .039, Cohen's *d*′ =  .201; the Block Design Test, *F* (1,322) = 4.23, *P* = .041, Cohen's *d*′ =  .229; the Beery-Buktenica Developmental Test of Visual-Motor Integration, *F* (1,338) = 4.10, *P* = .044, Cohen's *d*′ =  .220). Having cataracts also had a detrimental effect on performance (AAMR Adaptive Behavior Scale, *F* (1,425) = 20.44, *P* < .001, Cohen's *d*′ =  .439; the Block Design Test, *F* (1,322) = 20.78, *P* < .001, Cohen's *d*′ =  .508; the Beery-Buktenica Developmental Test of Visual-Motor Integration, *F* (1,338) = 12.55, *P* < .001, Cohen's *d*′ =  .385). Individuals with presbyopia, astigmatism, myopia, or strabismus performed comparably on all measures compared to individuals that did not have these conditions. For tasks that were independent of visual processing, the performance of individuals with and without an ophthalmic disorder and with or without any of the above specific ophthalmic conditions was comparable.

## 4. Discussion

The examination of medical records has shown that adults with Down syndrome are at an increased risk for ophthalmic disorders with advancing age. The chances of having at least one ophthalmic disorder increased significantly with age and older participants had a greater number of these disorders than younger participants. It was also clear that in adults with Down syndrome, specific ophthalmic disorders are closely related to the age of the individual. We found that while astigmatism and refractive errors were more prevalent in younger individuals, cataracts and blepharitis were more common in older individuals.

Contrary to previous studies, the prevalence of ophthalmic disorders was unrelated to severity of intellectual disability, with the one exception being that individuals who were legally blind were more likely to have profound intellectual disability. Given that visual processing is a relative strength for individuals with Down syndrome, this finding may reflect atypically severe consequences of visual impairment on cognitive development, but at this point it seems clear that valid interpretation will be dependent upon further investigation.

Cataracts were the most prevalent ophthalmic disorder recorded in medical charts for participants. As expected, prevalence increased with advancing age, and our data indicates that individuals with Down syndrome were significantly younger than individuals in the general population at the time of diagnosis [49]. This was consistent with an extensive body of literature documenting that people with Down syndrome show some signs of accelerated biological aging (e.g., [3, 51–53]). At the time of initial diagnosis, generally no treatment was prescribed for adults with Down syndrome and cataract surgery was reported infrequently. Many of the medical charts included a note that the condition was mild at the time of diagnosis and did not require treatment. We could not find comparable data on treatment prescribed at the time of diagnosis for adults in the general population, but further monitoring without immediate treatment is an accepted option within standard clinical practice.

As found in other studies, blepharitis and conjunctivitis, both inflammatory conditions of the eye, were found to be common conditions in individuals with Down syndrome. Blepharitis may be related to the narrow, slanted palpebral fissures characteristic in individuals with Down syndrome [54] or an increased susceptibility to infection associated with the impact of trisomy 21 on the immune system [22, 55, 56].

Severe visual impairment in adults without intellectual disability is known to negatively interfere with the ability to perform activities of daily living, especially those that rely on vision [57]. For example, difficulty with mobility [58, 59] and sleep problems [60] have been reported for older adults with low vision or blindness. Concerns about general safety may also come into play [53]. In individuals with intellectual disability, Evenhuis et al. [33] concluded that visual impairment compounds preexisting disability. We observed that in individuals with Down syndrome ophthalmic disorder(s) negatively affected adaptive behavior and cognitive functions that rely on visual processing. This was in contrast to the finding that individuals with and without ophthalmic disorder(s) performed comparably on selected skills that were independent of visual functioning (e.g., episodic memory and verbal fluency). We also observed that not all ophthalmic disorders were equally detrimental to adaptive behavior or cognition. Being legally blind had the most serious impact on participants' adaptive behavior skills and cognitive functioning, as one would expect, and having cataracts proved also to be detrimental. However, individuals with ophthalmic disorders were not affected to the extent that we expected. It is possible that ophthalmic disorders are being detected and treated appropriately in this population to a greater extent than previously supposed (cf. [61]), at least within networks serving our study participants.

 An important limitation of the present analysis is the reliance on data from medical charts. Medical charts can be inaccurate or incomplete compared to direct examination. For example, charts frequently made no mention of treatments prescribed for ophthalmic conditions, but that could be either because no treatments were provided or no notation of provided treatments were made.

Our results have important implications with respect to the ophthalmic care of adults with Down syndrome. The high prevalence of ophthalmic disorders highlights the need for periodic evaluations of adults with Down syndrome to identify age-related changes and other pathological eye conditions. In an IASSID International Consensus Statement, Evenhuis and Nagtzaam [62] proposed that planned vision screening and examinations for adults with Down syndrome should begin by age 30 and be conducted at least every five years. Pueschel et al. [63] and Van Buggenhout et al. [27] alternatively recommend more frequent assessments, at least every 2 years in adult patients with Down syndrome and increasing in frequency with advancing age. The present findings confirm the need for regular eye examinations, and the possibility of impaired vision needs to be investigated whenever declines in functional abilities occur in an older adult with Down syndrome.

## Figures and Tables

**Table 1:** Participant characteristics.

Characteristic	Down syndrome (*n* = 455)	
Age (Mean, SD)	(50.93, *7.85*)	

Computed FSIQ^1^ (Mean, SD)	(32.49, *9.37*)	

	*n*	%

Age group		
30–39	23	5.1
40–49	188	41.3
50–59	184	40.4
60–69	50	11.0
70–79	9	2.0
80+	1	.2

Level of intellectual disability		
Mild	30	6.9
Moderate	167	38.3
Severe	114	26.1
Profound	125	28.7

Sex		
Female	316	69.5
Male	139	30.5

Presence of ophthalmic disorders	353	77.6

^1^IQs were unavailable for 19 adults (4.2%).

**Table 2:** Common ophthalmic findings and percentage prevalence.

Ophthalmic conditions	
Amblyopia	13 (2.9%)
*Aphakia*	13 (2.9%)
Blepharitis	46 (10.1%)
Legal blindness	35 (7.7%)
Cataracts	191 (42.0%)
Conjunctivitis	61 (13.4%)
*Diabetic retinopathy*	0
*Dry eye*	4 (.9%)
Glaucoma	9 (2.0%)
Keratoconus	13(2.9%)
*Macular degeneration*	8 (1.8%)
Nystagmus	16 (3.5%)
Presbyopia/*hyperopia *	57 (12.5%)
*Pseudoaphakia*	11 (2.4%)
*Pterygium*	10 (2.2%)
*Ptosis*	3 (.7%)
Refractive error	115 (25.3%)
Astigmatism	52 (11.4%)
Myopia	88 (19.3%)
*Retinal detachment*	2 (.4%)
*Retinitis pigmentosa*	1 (.2%)
Strabismus	96 (21.1%)
Esotropia	79 (17.4%)
Exotropia	2 (.4%)

**Table 3:** Age-related prevalence of cataracts.

Age (years)	Down syndrome (%)	General population in United States without intellectual disability^1^
30–39	13.0%	—^2^
40–49	37.8%	2.5%
50–59	42.9%	6.8%
60–69	60.0%	20.0%
70–79	77.8%	42.8%
80+	100.0%^3^	68.3%

^1^The Eye Diseases Prevalence Research Group (2004a) [49] and summary data available at: http://nei.nih.gov/eyedata/pbd_tables.asp.

^2^Data unavailable.

^3^Only one participant in this age category.

**Table 4:** Adjusted least square means and standard errors for adaptive behavior and cognitive measures as a function of ophthalmic status.

Performance measure	With ophthalmic disorders	Without ophthalmic disorders
*Functions dependent on visual processing*		

AAMR-Adaptive Behavior Scale	169.12 (2.73)	179.81 (4.63)
Block Design Test	10.09 (.50)	12.71 (.79)
Beery-Buktenica Developmental Test of Visual-Motor Integration	8.28 (.24)	9.19 (.39)

*Functions independent on visual processing*		

Selective Reminding Test	23.11 (.82)	24.03 (1.27)
Verbal Fluency Test	5.66 (.24)	5.68 (.24)

## Appendix

### Definitions of Ophthalmic Terms

Amblyopia (Lazy Eye): Poor vision in one or both eyes that is not associated with any specific pathology and that persists after the correction of refractive errors.

Aphakia: Absence of the lens of the eye due either to surgical removal, a perforating wound or ulcer, or a congenital abnormality.

Astigmatism: Unequal curvatures along the different meridians in one or more of the refractive surfaces of the eye.

Blepharitis: Chronic inflammation of the eyelids.

Cataract: A clouding of the crystalline lens of the eye varying from a mild to complete opacity and resulting in the obstruction of the passage of light.

Conjunctivitis: Acute inflammation of the conjunctiva, the outermost layer of the eye and the inner surface of the eyelids. It is most commonly caused by an allergic reaction or an infection.

Cornea: The clear front window of the eyeball.

Diabetic Retinopathy: A condition, which causes progressive damage to the blood vessels of the retina resulting from complications of diabetes mellitus.

Dry Eye Syndrome (Keratitis Sicca): Chronic lack of lubrication and moisture in the eye.

Esotropia: A form of strabismus in which one or both eyes turns inward.

Exotropia: A form of strabismus in which one or both eyes are deviated outward. It is the opposite of esotropia.

Glaucoma: A group of diseases that damage the optic nerve and results in progressive and irreversible vision loss and blindness. It is frequently, although not always, associated with increased fluid pressure of the eye.

Hyperopia (Farsightedness): A refractive defect of the eye whereby near objects appear blurred because the image is focused in back of the retina rather than directly on it.

Keratoconus: A degenerative noninflammatory disorder of the eye in which structural changes within the corneal curve cause it to thin and subsequently to deform the shape of the cornea to a more conical shape from its normal gradual curve.

Myopia (Nearsightedness): A refractive defect of the eye whereby distant objects appear blurred because an image is focused in front of the retina, in the vitreous, rather than on it.

Nystagmus: Refers to the rhythmic, repetitive, oscillating, involuntary eye movements that occur when a large portion of the visual field moves constantly in a horizontal direction and can contribute to decreased vision. The movements consist of a slow phase in which the moving field is tracked (smooth pursuit), followed by a rapid “return” movement (saccade); this pattern is repeated until the field stops moving. In pathological cases, nystagmus can occur in the absence of a moving stimulus.

Optic Neuritis: Is an inflammation of the optic nerve that may result in a complete or partial loss of vision.

Presbyopia: Is the progressively diminishing ability to focus on nearby objects resulting from the loss of elasticity of the crystalline lens that occurs with advancing age.

Pseudoaphakia: A congenital condition in which the crystalline lens has degenerated and been replaced by mesodermal tissue.

Pterygium: Refers to a triangular thickening of the conjunctiva (outer coating of the eye) that grows onto the cornea causing redness, irritation, and tearing. If it grows large enough, it may interfere with vision.

Ptosis: Drooping of the upper eyelid in one or both eyes caused when the muscles that raise the eyelid (levator and Müller's muscle's) are not strong enough to do so properly.

Refractive Errors: Errors in the focusing of light, for example, myopia.

Retinal Detachment: A disorder of the eye in which the inner layers of the retina separate from the underlying layer of supportive tissue, the retinal pigment epithelium.

Retinitis Pigmentosa: A group of inheritable degenerative retinal diseases in which abnormalities of the photoreceptors (the rods and cones) or the retinal pigment epithelium led to progressive and incurable vision loss.

Strabismus: A condition in which the eyes are not properly aligned with each other. When looking at an object, the images do not fall on corresponding retinal locations.

Visual Acuity: Refers to a measure of the spatial resolving capacity of the visual system.
